# Myofascial Trigger Points Therapy Modifies Thermal Map of Gluteal Region

**DOI:** 10.1155/2020/4328253

**Published:** 2020-02-20

**Authors:** Grzegorz Onik, Teresa Kasprzyk, Katarzyna Knapik, Karolina Wieczorek, Dominik Sieroń, Aleksander Sieroń, Armand Cholewka, Karolina Sieroń

**Affiliations:** ^1^Medical University of Silesia in Katowice, School of Health Sciences in Katowice, Department of Physical Medicine, Chair of Physiotherapy, Katowice, Poland; ^2^A. Chełkowski Institute of Physics, Department of Medical Physics, University of Silesia, Katowice, Poland; ^3^Institute of Radiology and Neuroradiology, Tiefenau Hospital, Bern, Switzerland; ^4^Jan Dlugosz University, Czestochowa, Poland

## Abstract

**Background:**

Thermal imaging may be effectively used in musculoskeletal system diagnostics and therapy evaluation; thus, it may be successfully applied in myofascial trigger points assessment.

**Objective:**

Investigation of thermal pattern changes after myofascial trigger points progressive compression therapy in healthy males and females.

**Methods:**

The study included 30 healthy people (15 females and 15 males) with age range 19–34 years (mean age: 23.1 ± 4.21). Thermograms of myofascial trigger points were taken pre- and posttherapy and consecutively in the 15^th^ and 30^th^ minutes. Pain reproducible by palpation intensity was assessed with numeric rating scale.

**Results:**

Progressive compression therapy leads to myofascial trigger points temperature (*p*=0.02) and surface (*p*=0.02) and surface (*p*=0.02) and surface (*p*=0.02) and surface (

**Conclusions:**

The study indicates that myofascial trigger points reaction to applied therapy spreads in time and space and depends on participants' sex.

## 1. Introduction

Myofascial pain syndrome is a common chronic musculoskeletal condition caused by myofascial trigger points [1, 2]. They are defined as tight bands in a contracted muscle that can produce local and referred pain as well as sensory, motor, and autonomic symptoms [3, 4]. Clinically, myofascial trigger points can be identified by palpation and symptoms analysis, and thus classified as active or latent. Active myofascial trigger points display pain spontaneously, whereas in the latent ones pain reproducible by palpation occurs [1, 4, 5]. Myofascial trigger points may develop due to acute or repetitive muscle injuries or overloading, joints injuries, enthesopathies, spine and intervertebral disc pathologies, poor posture, and systemic diseases such as fibromyalgia [4–7].

Even though the pathophysiology of myofascial trigger points is not fully understood, the hypothesis of acetylcholine leakage is mostly accepted. Indeed, leakage of acetylcholine leads to impairment of sarcoplasmic reticulum with following extensive calcium release, as well as secondary sarcomere contraction and cell membrane damage. It results in local vasoconstriction leading to ischemia and hypoxia [3, 4]. That is why acetylcholine leakage at the neuromuscular junction may be significant in the context of thermal imaging [3].

Mostly in physiotherapeutic practise, myofascial trigger points are diagnosed with clinical symptoms analysis and manual examination [4], whereas available reports confirmed the diagnostic value of ultrasound imaging. Although ultrasound examination requires high-cost equipment as well, its sensitivity and specificity for detecting myofascial trigger points are still uncertain [8]. Those modalities should be accessible and cheap and provide reliable and easy interpretative measurements.

Following constant technological development and researches it is reasonable to apply thermal imaging in myofascial trigger points diagnostics. Infrared thermography is a noncontact and noninvasive technique applied in subjects with a temperature higher than absolute zero (0 K). It enables body surface temperature distribution and changes processing [9]. The analysis of skin temperature distribution may indicate subcutaneous blood perfusion changes, which correlate with local or global physiological conditions [10, 11]. Therefore, infrared thermography is willingly applied in different medical specialties [12].

We hypothesize that the application of progressive pressure technique leads to thermal pattern changes in infrared thermography. The main aim of the study was to investigate thermal pattern changes after myofascial trigger points progressive compression therapy in healthy males and females.

## 2. Materials and Methods

The study sample size was set with the usage of *G* Power 3.1.94 software. With the assumption of effect size ƒ = 0.45, *α* = 0.05 and statistical power (1-*β* error probability) = 0.85, the sample size was established on 30 people, showing an actual power of 0.852.

The study included 30 healthy people (15 females and 15 males) with age range 19–30 years (mean age: 23.1 years  ± 4.21 years). Participants' mean height was 1.74 m ± 0.08 m while the mean weight was 66.86 kg ± 12.18 kg. The study group mean body mass index was 21.65 kg/m^2^ ± 2.65 kg/m^2^. Participants' anthropometric characteristics with division into sex are presented in Table 1.

Participants had to meet the following inclusion criteria: the presence of latent myofascial trigger points in the gluteal area, consent agreement, and age between 18 and 30 years. The exclusion criteria were as follows: neuromuscular diseases, gluteal region traumas, nonsteroidal anti-inflammatory, opioid, anticoagulant, and antiplatelet drug intake two weeks prior to the study, superficial sensation impairment, severe low back pain, menarche, pregnancy, acute inflammation, and BMI ≥ 30 kg/m^2^.

To ensure study reliability thermal imaging was performed in a test room with constant temperature (22°C ± 1°C) and humidity (40–45%); subjects were instructed to refrain from applying lotions and creams one day before the study. To prevent cloths-mediated friction and following skin temperature increase, participants had to pass an adaptation process before the thermal imaging. The adaptation process included 15 minutes of rest with the uncovered gluteal area. The thermograms of regions of interest were taken with Flir Systems E60 thermal camera, with 0.05 K sensitivity. The distance between participants' bodies and the thermal camera was set at 1 meter. Thermograms were taken four times: just before, immediately after the progressive compression therapy, and consecutively in the 15^th^ and 30^th^ minutes.

Progressive compression therapy of each myofascial trigger point lasted 1 minute and was performed by the same physiotherapist. Myofascial trigger points were assumed to overlap with regions of increased temperature in comparison to the surrounding tissues. What is more, myofascial trigger points location was confirmed by a preliminary palpation one day before the thermal imaging. The thermograms were compared with the point marked on the subjects' bodies. The myofascial trigger points size was measured as a number of pixels. Moreover, pain reproducible by palpation was assessed in participants with Numeric Rating Scale before and after the therapy. The isotherm function was applied to perform thermal images analysis. The level of isotherm was set on 32.60°C. This value showed all regions of interest.

During this cross-sectional descriptive study, the Helsinki Declaration and ethical standards in human experimentation were respected. The study protocol was approved by the Bioethics Committee of the Medical University of Silesia in Katowice, Poland.

Statistical analysis was performed with STATISTICA 13 software. Data distribution was checked with the Shapiro-Wilk test. If data distribution was normal *t*-Student test was used. Unless data were distributed normally, *U* Mann-Whitney test was applied. Analysis of variance was performed with Levene's test. If homogeneity of variance was confirmed one-way ANOVA with post hoc Tukey was used. Correlations were calculated with Pearson correlation coefficient. The relationships between pain intensity value and myofascial trigger points temperature during the pretherapy measurement were evaluated by linear regression analyses. Statistical significance level was set at *p* < 0.05.

## 3. Results

In all participants, the temperature of myofascial trigger points was increasing in consecutive measurements until the 15^th^ minute whereas in the 30^th^ minute the temperature decrease was observed (*p*=0.02). In the 15^th^ minute, the temperature was significantly higher than in pretherapy measurement (*p*=0.04). Comparison of pre- and posttherapy myofascial trigger points mean temperature between males and females did not reveal significant differences. Myofascial trigger points mean temperature in males was growing in consecutive measurements (*p*=0.02). In comparison to pretherapy myofascial trigger points mean temperature in the 15^th^ (*p*=0.048) and 30^th^ (*p*=0.04) minute was significantly higher (Table 2), whereas in females the mean temperature of myofascial trigger points did not differ significantly.

Pain reproducible by palpation after the application of progressive compression therapy decreased significantly in all participants (*p*=0.01). With regard to the sex pain intensity decrease was also observed: males (*p*=0.03) and females (*p*=0.048). Between the sexes, there were no significant differences in pain intensity in the assessed period of time (Table 3).

In all participants, the surface of myofascial trigger points was increasing in consecutive measurements until the 15^th^ minute whereas in the 30^th^ minute the decrease was observed (*p* ≤ 0.001). In the 15^th^ and 30^th^ minutes myofascial trigger points surface was significantly higher than in pretherapy measurement (*p*=0.001). Comparative analysis of myofascial trigger points surface did not reveal significant differences between males and females in consecutively taken thermograms. Pressure application in males led to a significant increase of myofascial trigger points size (*p*=0.01). In comparison to pretherapy size, the surface of myofascial trigger points increased significantly at thermograms taken in the 15^th^ (*p*=0.03) and 30^th^ (*p*=0.02) minutes. Whereas in females the mean surface of myofascial trigger points did not differ significantly (Table 4).

In all participants before therapy, statistically significant negative correlation (*r* = 0.39, *p* < 0.05) between myofascial trigger points temperature and pain intensity was observed. In males, similar results were obtained (*r* = 0.6, *p* < 0.05). Higher temperature was correlated with lower pain. The remaining correlations were not statistically significant (Table 5).

**Figure 1:**
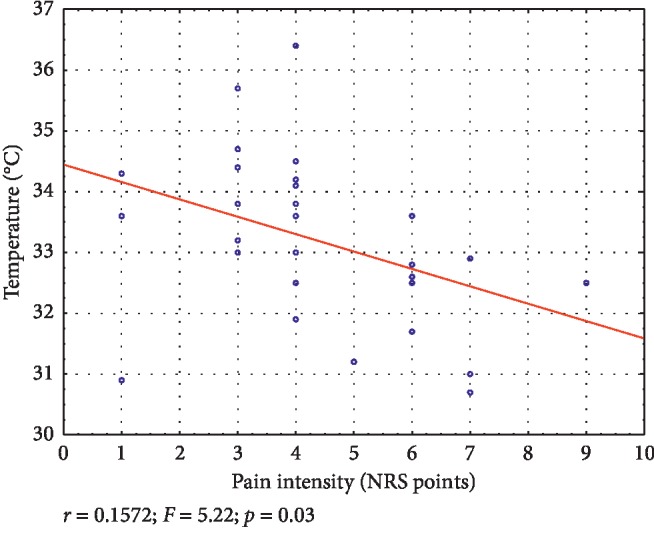
Linear regression model for temperature and pain intensity during pretherapy measurement in all participants.

**Figure 2:**
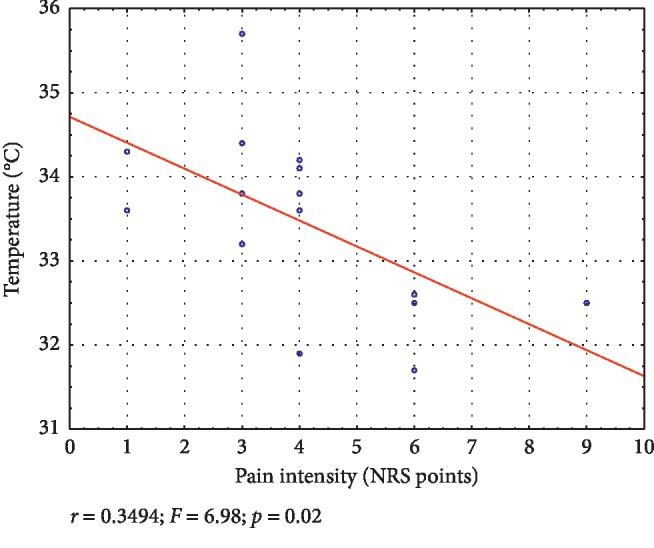
Linear regression model for temperature and pain intensity during pretherapy measurement in males.

Regression models during the first measurement for temperature and pain intensity are presented in Figures 1 and 2. In the whole study group, a higher temperature was related to lower pain intensity. With regard to sex, similar results were obtained in males. Regression models for the remaining variables (age, body mass index, myofascial trigger points surface) were not statistically significant. What is more, regression models performed between the study stages were not statistically significant.

## 4. Discussion

To our knowledge, this is the first study evaluating the behaviour of myofascial trigger points thermal patterns after applied therapy. The main finding of the study is the establishment of therapy-mediated myofascial trigger points temperature increase prolongation in consecutive thermograms and what is more, temperature changes reveal sex dependency.

Thermal imaging during the myofascial trigger points therapy showed that the average temperature of the region of interest was increasing after the progressive pressure therapy in males and females. Analysis of thermal images with the usage of isotherms showed a significant increase of surface temperature above 32.6°C. Warmer area increase may result from physiological changes mediated by progressive compression, which is a frequently applied technique in myofascial trigger points treatment. The pressure application leads to occlusion of local blood vessels and hypoxia strengthening [13, 14]. After the therapy, extensive vasodilatation occurred, and thus warmed area surface increased. It might result from nitric oxide release, which plays an important role in intramuscular vasodilatation [15, 16]. The hypothesis of integration postulates an additional acetylcholine leakage in the neuromuscular junction leading to persistent sarcomere contraction with the following vasoconstriction and tissue ischemia [5]. However, potential acetylcholine binding with muscarinic receptors, especially with subtypes *M*_1_, *M*_3_, and *M*_5,_ may also reflect on posttherapy temperature increase as those receptors mediate endothelium-dependent vasodilation [5, 17, 18]. What is more, the presence of histamine, substance P, and bradykinin in the surrounding tissues may additionally enhance posttherapy vasodilatation [5]. Moreover, heat exchange between neighbouring blood vessels may also be responsible for myofascial trigger points' surface increase obtained in thermograms [19].

Posttherapy extensive increase of myofascial trigger points surface, which were primarily established to be the overheated regions, may also be associated with the second law of thermodynamics stating that heat transfer always occurs from a higher temperature object to a cooler-temperature one. It may also reflect on myofascial trigger points' size as heat might be conducted to surrounding tissues [20]. Nevertheless, heat transfer between the physiotherapist's finger and participants' skin should be also taken into consideration. Heat conduction happens if bodies contact directly; that is why an observed posttherapy temperature increase might be a consequence of therapy-mediated activation of physiological processes as progressive compression therapy lasted only 1 minute [20].

Myofascial trigger points treatment may be treated as two-phase consisting of pain-control and deep conditioning phases [21]. Anterograde character of treatment as well as the obtained results indicating myofascial trigger points temperature and surface increase in time makes it reasonable to evaluate therapy effectiveness with adequate delay.

Females, compared to males, have a higher percent of body fat and deposit it in a different pattern, with relatively more adipose tissue in the gluteofemoral area [22]. It might explain the lower temperature of myofascial trigger points in females at every stage of observation. The amount of fatty tissue in the gluteal region may impair the heat transfer as adipose tissue affects the blood flow regulation. What is more, it may explain myofascial trigger points temperature decrease in the 30^th^ minute posttherapy in females [23, 24]. It proves temperature gradient alignment and thermal equilibrium reaching as well [20]. In further studies dedicated to myofascial trigger points thermal imaging, it would be reasonable to provide more information about participants' body composition based on, for example, bioelectrical impedance analysis [25].

Some researchers state that hot spots do not correspond with myofascial trigger points localization and infrared thermography cannot be applied in their identification because of thermal pattern absence [26, 27]. However, a study protocol supplementation with pain intensity assessment increases the study's reliability and confirms myofascial trigger points detection with infrared thermography.

Pretherapy pain intensity was higher in females. This data stays in line with available literature indicating a higher prevalence and pain intensity in women [28]. Peripheral nociceptors sensitization may result from the secretion of bradykinin, substance P, and serotonin [21]. As those agents may mediate posttherapy extensive vasodilatation and improve blood flow they may also augment pain reduction [5]. In male and female participants therapy-mediated pain reduction occurred. Observed myofascial trigger points temperature increase refers to improved circulation as a consequence of vasocongestion. Blood flow properties enhancement and secondary proinflammatory cytokines leaching may explain pain reduction in the study group [14].

Therapy-mediated thermal effects in myofascial trigger points may indicate physiological processes being secondary to the applied treatment. Infrared thermography may be utile in localization and evaluation of myofascial trigger points because of its noninvasive character and real-time measurement. It may provide valuable information for physicians or physiotherapists as well as allow for the objectification of myofascial trigger points diagnosis [29].

The negative correlation between superficial temperature and pain intensity may be explained by sufficient blood flow preventing ischemia and secondary pain [30]. However, Wu et al. claim that skin temperature variations have little impact on pain sensitivity [31]. Gutiérrez-Rojas et al. found that the application of ice leads to a rapid change of pressure pain perception in patients with latent myofascial trigger points in supraspinatus muscle [32]. Despite the fact that the application of cold decreases pain intensity mainly by reduction of nociceptive conduction velocity in peripheral nerves it also impairs local blood flow [33]. Progressive pressure therapy results in constriction of blood vessels that enhances local hypoxia [13, 14]. The linear regression model revealed that lower pain intensity mediates a higher temperature of myofascial trigger points. Increased blood perfusion in myofascial trigger points observed during the pretherapy measurement may be a compensatory mechanism being the reaction to hypoxia resulting from sarcomere contracture [3, 4]. Blood flow enhancement may explain decreased pain intensity as proinflammatory agents are flashed out [14]. On the other hand, a higher temperature may indicate the presence of local inflammation which is characterized by vascular dilation, enhanced permeability of capillaries, and increased blood flow [34]. In that way, the application of cold may provide some desirable effects. As the application of cold and progressive pressure therapy is followed by excessive vasodilatation, in further studies it would advisable to assess potential risk and presence of reperfusion injury by measurement of creatine kinase level [33].

Except for progressive pressure technique in the treatment of myofascial trigger points various methods may be applied, such as physical therapy modalities, dry needling, or myofascial trigger points injection [35]. Benito-de-Pedro et al. compared the thermal pattern changes after deep dry needling and ischemic compression in latent myofascial trigger points suggesting that ischemic compression might be more advisable [36]. This may support the legitimacy of progressive pressure technique usage in our study.

Our study has some limitations. Firstly, the study included healthy subjects with inactive myofascial trigger points. However, this enabled us to investigate the thermal pattern changes. Secondly, myofascial trigger points location was set with manual palpation. Despite the fact that manual examination has limitations it is the most popular method of nodules detection [8]. Thirdly, we were unable to assess the applied pressure value. In further studies, objective methods of pressure application should be used to determine the influence of pressure value on thermal response. Fourthly, we did not assess the potential influence of musculoskeletal system disorders on the thermal map of the human body. Rodríguez-Sanz et al. found that thermal pattern changes may be a consequence of ankle equinus [37]. That is why in further studies assessment of body posture and inspection for compensatory mechanisms should be performed prior to measurements. Finally, our study was aimed at the assessment of latent myofascial trigger points with infrared thermography. As Calvo-Lobo et al. confirmed the utility of sonoelastography in the assessment of active and latent myofascial trigger points, further studies might be implemented with that modality [38].

Summing up, our study indicates that myofascial trigger points reaction to applied therapy spreads in time and space. It may be an important clinical implication that will facilitate the adequate evaluation of myofascial trigger points therapy effectiveness with reference to the sex. What is more, it also proves that physiotherapists should perform posttherapy evaluation with adequate delay. Immediate therapy effects refer mainly to pain intensity whereas tissue response is a long-lasting process.

## Figures and Tables

**Table 1:** Characteristics of participants.

	Males	Females	*p* value
Age (years) (M ± SD)	23.8 ± 5.17	22.4 ± 2.78	0.84
Weight (kg) (M ± SD)	73.73 ± 12.43	60 ± 6.19	**0.0005**
Height (m) (M ± SD)	1.78 ± 0.08	1.7 ± 0.07	**0.002**
BMI (kg/m^2^) (M ± SD)	23.09 ± 2.77	20.81 ± 1.81	**0.008**

**Table 2:** Therapy-mediated myofascial trigger points temperature changes.

	Mean temperature of myofascial trigger points (°C)				ANOVA (*p* value)	Post hoc Tukey (*p* value)	
	Before therapy (A)	After therapy (B)	15 minutes after therapy (C)	30 minutes after therapy (D)			
All	33.21 ± 1.36	33.54 ± 1.23	34.1 ± 1.22	34.08 ± 1.43	**0.02**	A < B	0.75
						**A < C**	**0.04**
						A < D	0.06
						B < C	0.35
						B < D	0.4
						C < D	1.00

Males	33.46 ± 1.03	33.76 ± 1	34.51 ± 0.95	34.52 ± 1.21	**0.02**	A < B	0.86
						A < C	0.05
						**A < D**	**0.04**
						B < C	0.25
						B < D	0.24
						C < D	1.00

Females	32.95 ± 1.49	33.28 ± 1.31	33.7 ± 1.25	33.63 ± 1.41	0.49	—	

ΔT1 (°C)	0.51	0.43	0.81	0.89	—	—	
*p* value	0.31	0.34	0.07	0.09	—	—	

Note: ^1^temperature difference between males and females.

**Table 3:** Therapy-mediated pain intensity changes.

	Mean intensity of pain reproducible by palpation		*p* value^2^
	Before therapy (A)	After therapy (B)	
All	4.33 ± 1.88	3.07 ± 1.82	**0.01**
Males	4.07 ± 1.98	2.73 ± 1.49	**0.03**
Females	4.6 ± 1.67	3.4 ± 1.99	**0.05**
*p* value^1^	0.22	0.16	

Note: ^1^pain intensity difference between males and females; ^2^pain intensity difference between measurements.

**Table 4:** Therapy-mediated myofascial trigger points surface changes.

	Mean surface of myofascial trigger points (px)				ANOVA (*p* value)	Post hoc Tukey (*p* value)	
	Before therapy (A)	After therapy (B)	15 minutes after therapy (C)	30 minutes after therapy (D)			
All	504.6 ± 583.41	1135.93 ± 1139.88	1764 ± 1628.42	1681.3 ± 1513.41	*p* < 0.001	A < B	0.23
						**A < C**	**0.001**
						**A < D**	**0.001**
						B < C	0.24
						B < D	0.36
						C < D	0.99

Males	484.87 ± 530.82	1132.6 ± 1055.03	2073.4 ± 1948.58	2140.4 ± 1849.39	**0.01**	A < B	0.65
						**A** < **C**	**0.03**
						**A** < **D**	**0.02**
						B < C	0.36
						B < D	0.28
						C < D	0.99

Females	524.33 ± 612.28	1139.3 ± 1182.77	1454.6 ± 1066.9	1222.2 ± 765.74	**0.07**	—	—

ΔS1 (px)	39.47	6.67	618.8	918.2	—	—	—

*p* value	0.98	0.95	0.45	0.18	—	—	—

^1^myofascial trigger points difference between males and females.

**Table 5:** Correlations before and after therapy in males and females.

Temperature (°C)	Pearson's correlation (*r*)			
	Age (years)	Weight (kg)	Surface (px)	Pain intensity (NRS points)
All before therapy	−0.25	−0.09	0.06	−0.39^*∗*^
All after therapy	−0.14	−0.15	0.07	0.06
Males before therapy	−0.4	−0.38	0.1	−0.6^*∗*^
Males after therapy	−0.21	−0.48	0.14	−0.31
Females before therapy	−0.24	−0.17	−0.05	−0.23
Females after therapy	−0.18	−0.16	0.03	0.31

Note: ^*∗*^*p* < 0.05.

## Data Availability

The data used to support the findings of this study are made available from the corresponding author upon request.
